# The Generation of Insulin Producing Cells from Human Mesenchymal Stem Cells by MiR-375 and Anti-MiR-9

**DOI:** 10.1371/journal.pone.0128650

**Published:** 2015-06-05

**Authors:** Arefeh Jafarian, Mohammad Taghikani, Saeid Abroun, Amir Allahverdi, Maryam Lamei, Niknam Lakpour, Masoud Soleimani

**Affiliations:** 1 Department of Clinical Biochemistry, Faculty of Medical Sciences, Tarbiat Modares University, Tehran, Iran; 2 Department of Hematology, Faculty of Medical Sciences, Tarbiat Modares University, Tehran, Iran; 3 Nanobiotechnology Research Center, Avicenna Research Institute, ACECR, Tehran, Iran; University of Torino, ITALY

## Abstract

**Background:**

MicroRNAs (miRNAs) are a group of endogenous small non-coding RNAs that regulate gene expression at the post-transcriptional level. A number of studies have led to the notion that some miRNAs have key roles in control of pancreatic islet development and insulin secretion. Based on some studies on miRNAs pattern, the researchers in this paper investigated the pancreatic differentiation of human bone marrow mesenchymal stem cells (hBM-MSCs) by up-regulation of miR-375 and down-regulation of miR-9 by lentiviruses containing miR-375 and anti-miR-9.

**Methodology:**

After 21 days of induction, islet-like clusters containing insulin producing cells (IPCs) were confirmed by dithizone (DTZ) staining. The IPCs and β cell specific related genes and proteins were detected using qRT-PCR and immunofluorescence on days 7, 14 and 21 of differentiation. Glucose challenge test was performed at different concentrations of glucose so extracellular and intracellular insulin and C-peptide were assayed using ELISA kit. Although derived IPCs by miR-375 alone were capable to express insulin and other endocrine specific transcription factors, the cells lacked the machinery to respond to glucose.

**Conclusion:**

It was found that over-expression of miR-375 led to a reduction in levels of Mtpn protein in derived IPCs, while treatment with anti-miR-9 following miR-375 over-expression had synergistic effects on MSCs differentiation and insulin secretion in a glucose-regulated manner. The researchers reported that silencing of miR-9 increased OC-2 protein in IPCs that may contribute to the observed glucose-regulated insulin secretion. Although the roles of miR-375 and miR-9 are well known in pancreatic development and insulin secretion, the use of these miRNAs in transdifferentiation was never demonstrated. These findings highlight miRNAs functions in stem cells differentiation and suggest that they could be used as therapeutic tools for gene-based therapy in diabetes mellitus.

## Introduction

Diabetes mellitus is a metabolic disorder affecting 2–5% of the population. Transplantation of isolated islets of Langerhans from donor pancreata could be a cure for diabetes. However, such an approach is limited by the scarcity of the donors and the long term, significant side effects of immunosuppressive therapy. Using a renewable source of cells, such as different kinds of stem cells may be an efficient way for overcoming these problems [1]. In 2001, human embryonic stem (hES) cells were reported to have the capacity to generate IPCs by spontaneous differentiation in vitro [2]. MSCs differentiation to pancreatic islet cells was first reported in 2004. MSCs have great multiplication potency, cell doubling time is 48–72 h and cells could be expanded in culture for more than 60 doublings [3]. These cells have immunoregulatory properties and do not elicit immune response [4]. Histological studies on these cells in comparison with ESC have not shown any tumor formation after transplantation. The use of adult stem cells will circumvent the ethical dilemma surrounding embryonic stem cells and will allow autotransplantation [5]. MSCs are mostly advantageous for experimental use. They are usually collected from different adult stem cell sources, purified and used therapeutically and they also possess the potential for treatment of type 1diabetes (T1DM) through autologous procedure. miRNAs are a novel class of endogenous small nc-RNAs, of ~20–30 nucleotides in length that were first discovered in 1993 in Caenorhabitis elegans and Drosophila and later identified in many species [6]. These nc-RNAs are encoded by up to 3% of all genes and approximately 30% of the genes are supposed to be regulated by small RNA species that regulate gene expression post-transcriptionally [7–10]. In mammalians, miRNAs have inhibitory effects on RNA stability and mRNA translation by base pairing in 3^'^ untranslated regions (UTRs) of target mRNAs [11]. Recent discoveries have identified several miRNAs that have potential roles in pancreas development, islet function, insulin secretion and diabetic complications [12, 13]. miR-375 has been identified as a highly expressed miRNA in pancreatic islets which is involved in islet development [14], control of insulin gene expression and secretion [15]. Targeted inhibition of miR-375 in zebrafish resulted in major defects in pancreatic development and aberrant formation of the endocrine pancreas [14]. On the other hand, investigations revealed that miR-375 has inhibitory role in glucose-stimulated insulin secretion (GSIS) [16] through targets myotrophin (Mtpn), a protein involved in insulin granule fusion [15, 17]. miR-9 is another miRNA that has been involved in the control of insulin exocytosis by targets Onecut-2 (OC-2) mRNA and down regulates its expression in insulin producing cells. Thus the observed decrease in OC-2 expression may lead to an increase in the levels of its target gene, granuphilin. Granuphilin has been well characterized as a negative regulator of insulin secretion [18].Several studies have been carried out to generate insulin producing cells (IPCs) from ESCs or various adult stem cells using different combination of growth factors and cytokines cocktail [2, 19]. However, the main problem of these methods during regenerative therapies in large scale is repeatability error and high cost. On the other hand, a number of studies have led to the notion that some miRNAs have key roles in control of pancreatic islet development and insulin secretion [20]. Hence, according to some studies on microRNA pattern, expression modulation of certain miRNAs may possibly be a useful method for islet-like aggregates differentiation. So, in this study, the researchers examined whether up-regulation of miR-375 and down-regulation of miR-9 could induce functional islet-like cellular aggregates differentiation in MSCs derived from human BM. The obtained results indicate that miR-375 up-regulation could be an effective factor for in vitro differentiation of hMSCs into islet-like cellular aggregates and might be the initial step in producing pancreatic islets by means of miRNAs. However, these cellular aggregates didn’t have any response to different concentrations of glucose in vitro. On the other hand, down-regulation of miR-9 after induction into islet-like aggregates by lentivirus containing miR-375 could significantly increase cellular response to different concentrations of glucose by increasing OC-2 protein expression that may consequently lead to a decrease in levels of its target gene, granuphilin, which has negative role in insulin secretion. It is expected that this novel method can be used as a therapeutic option for diabetic patients.

## Materials and Methods

### Cell purification and culture of human BM-MSCs

All the samples were obtained by a physician after receiving written informed consent and permission from the local ethics committees at Taleghani Hospital and Tarbiat Modares University (Tehran, Iran) (permission number; 112234). Heparinized BM samples were isolated from the posterior superior iliac crest of five healthy human (n = 5), aged 20–54 years as previously described [21]. In Brief, whole bone marrow was diluted at 1:1 ratio with phosphate buffered saline (PBS, PH = 7.2).Subsequently mononuclear cells fractions were obtained using a density gradient (ficoll-paque; 1.073g/ml, Pharmacia Uppsala, Sweden), the cells were cultured in 5 ml of Dulbecco’s Modified Eagle’s Medium (DMEM) (Gibco, Germany) supplemented with 10% fetal bovine serum (Gibco) and100u/ml penicillin/streptomycin (Gibco), incubated at 37°c for 72 hrs under a 5% CO2 condition to obtain adherent cells. Non-adherent cells were removed and adherent cells were maintained in complete medium for 2 weeks. The culture medium was replaced twice a week. After reaching nearly 90–95% confluent cells, the cells were trypsinized with 0.25% trypsin and 1mM EDTA (Sigma, St. Louis, Mo) and then MSCs were cultured in 75 cm^2^ flasks to achieve the required confluence.

### Flow cytometric analysis of human BM-MSCs

Human BM-MSCs at passage 3 were detached by trypsinization. The cells were incubated with labeled phycoerythrin (PE)-conjugated monoclonal antibodies at the dilution indicated by the manufacturer at 4°c for 30 minutes in the dark. The antibodies used were anti-human CD105-PE, CD90-PE, CD13-PE, CD34-PE (BD Biosciences, USA) and PE-labeled isotype-matched immunoglobulin was used as a negative control. The labeled cells were analyzed on a FACSCaliber (Becton-Dickinson, FAC scan, San Jose, CA, USA)

### Characterization of human BM-MSCs

hBM-MSCs at passages 3–5 were used to induce differentiation into adipocytes and osteocytes. The cells were plated at 2×10^4^ cells/well in a 24-well plate in DMEM supplemented with 10nM dexamethasone, 0.5μg/ml insulin, 2mM glutamine, 10% FBS and antibiotics. The medium was changed twice a week. After 2 weeks, the cells were fixed with 4% paraformaldehyde (PFA) for 20 minutes and then incubated with lipid vacuoles stained with Oil-Red-O stain (Sigma) for 15 minutes. Adipogenic differentiation was observed under inverted phase contrast microscope. For osteoblastic differentiation, hBM-MSCs were seeded at 1X10^4^ cells/well in a 24-well plate. The cells were cultured in L-DMEM supplemented with 10% FBS, 10^−8^ M dexamethasone (Sigma), 10mM β-glycerol phosphate (Sigma) and 50mg/ml ascorbic acid-2 phosphate (Sigma) for 21 days. Then the cells were fixed in 4% paraformaldehyde for 20 minutes and stained with alizarin red stain for 10–15 minutes. The production of intracellular calcium deposition that was observed under inverted phase contrast microscope was indicator of osteogenic differentiation.

### Lentiviral production

The lentivirus miR-375 and anti-miR-9 were generated from the co-transfection of 70–80% confluent HEK 293T cells with lentiviral packaging plasmids, *psPAX2* (containing *gag* and *pol* genes), *pMD2*.*G* (containing VSV-G gene) and *hsa-mir*-375 (containing the CMV and SV40 promoters) (Applied Biological Materials, Canada, mh10566) *inhibitor hsa-III-miR-9-off* (abm.mh31191) vector and *pLenti-III-mir-GFP-Blank* (abm.m001) empty vector that were co-transfected with lipofectamin 2000 transfection reagent (Invitrogen, USA) according to the manufacturer’s instructions. 48 hrs later, the lentiviral particles in supernatant were collected and filtered through a 0.45 mm filter and concentrated by ultracentrifuge at 35.000g for 2 hrs.

### Human BM-MSCs transduction

The hBM-MSCs at passage 3 with 70–80% confluences were used for transduction. Cells were plated at a density of 1X10^6^ cells/well in six-well plates. The study was performed in four groups; one group of cells was transduced with *hsa-miR*-375 lentiviruses carrying GFP (MSCs^miR-375^), another was infected with *hsa-miR-9-off* lentiviruses carrying GFP (MSCs^anti-miR-9^) (in this group the cells were briefly infected with *hsa-miR-375* lentivirus and 7 days later the cells were exposed to *hsa-miR-9-off* lentivirus), the third was infected with both miRNAs (MSCs^miR-375+anti-miR-9)^) and the forth group was transduced with *pLenti-empty* lentiviruses carrying GFP (MSCs^null^). The researchers used control MSCs (MSCs^control^) without any treatment to compare the effects of empty vectors without carrying miR-375 and miR-9 on cell lineage decisions. The cells were transduced with miR-375 and/or miR-9 lentivirus and empty virus at a multiplicity of infection (MOI) of 10. After selection by 2μg/ml puromycin, the cells were cultured in fresh complete serum-free medium containing DMEM (Gibco) supplemented with 1.5% BSA (A8806, Sigma) in the absence of any β cell stimulatory growth factors and cytokines for 21 days. 3 days after transduction, the efficiency of infection was evaluated by observation of GFP expression under a fluorescence microscope (Nikon.US).

### Measuring the expression levels of miR-375 and miR-9 by qRT-PCR

The researchers used the same tracer (GFP) for MSCs^miR-375^ and MSCs^anti-miR-9^. In order to confirm the transduction efficiency and the observation of GFP expression under a fluorescence microscope, qRT-PCR technique was used. Total RNA was extracted from 1×10^6^ transduced hMSCs on day 4 with Trizol reagent (Invitrogen) according to the manufacturer’s instructions and 5 ng of total RNA was reverse transcribed by mercury LNA universal cDNA synthesis kit (Exiqon, Denmark, Product No: 203300) at 42°c for 60 min and at 95°c for 5 min. Quantitative real-time PCR was prepared in triplicate measurements of total 10 μl reaction with a cDNA at a dilution of 1:80 and mercury LNA SYBR Green master mix (Exiqon, Denmark, Product No: 203450) according to the manufacturer’s instructions with has-miR-375 (Exiqon, Product No: 204362), has-miR-9 (Exiqon, Product No: 204513) LNA PCR primers, and U6 small nuclear RNA endogenous control primer (Exiqon, Product No: 203907). The qRT-PCR cycling conditions were 10 minutes at 95°C followed by 10 sec at 95°C and 1min at 60°C repeated for 45 step cycles. Data analyses were performed using the relative quantification of CT approach.

### Dithizone staining

DTZ staining was used to evaluate the formation of spheroid islet-like cluster containing IPCs on day 14 after induction. For producing stock solution that was previously described [22], 50mg of DTZ (Sigma) was completely dissolved in 5ml of dimethyl sulfoxide (DMSO, Sigma) and then it was stored at 20°c in dark. For the staining, the working solution was prepared by diluting the stock solution (PH 7.8) 1:100 in culture medium. 3ml of DTZ working solution was added into each well and incubated for 30 minute at 37°c. Then crimson- red differentiated clusters were observed under an inverted phase contrast microscope.

### Cell viability assay

Cell viability assay was employed to detect the survival rate of differentiated cells by assaying the reduction of 3-(4,5-dimethylthiazol-2-yl)-2,5-diphenyltetrazolium bromide (MTT) to formazan that was previously reported [23]. For this purpose, on days 7, 14 and 21 post infections, cells were seeded at the density of 1×10^5^ cells/well in a 96-well tissue culture plate and incubated for next day at 37°c. Then 20μl MTT (0.5mg/ml) (Sigma) was added to each well and plates were incubated at 37°c for 4hrs in dark. After incubation, supernatant was discarded and formazan crystals were dissolved by adding 0.1ml of DMSO in each well. Absorbance of each well was measured by ELISA-Reader (Biochrom Anthos 2020, UK) at 570nm and a reference wavelength of 630 nm to gain sample signal (OD570—OD630). The results were expressed as the mean ± SEM that was reported at least three times.

### RNA extraction and qRT-PCR

Total RNA was isolated from test and control cells on days 7, 14 and 21 post infection using Trizol (Invitrogen) according to the manufacturer’s instructions. The first strand cDNA was synthesized with 50 ng of total RNA by random hexamer priming using high capacity cDNA synthesis kit (iNtRON, Korea) at 42°c for 60 min and at 70°c for 5 min. Quantitative real-time PCR was performed with a SYBR-Green kit (Takara, Korea) according to the manufacturer’s instructions and using the ABI Light Cycler (ABI step one) in a total reaction volume of 10μl. All reactions were performed in triplicate and results were normalized to GAPDH (internal control) to correct RNA input in reactions. The primers were designed using AlleleID software (Primer Biosoft) which is listed in Table 1. All reactions were performed by annealing at 60°c for 40 cycles and the melt curve analysis was achieved at the end of each reaction. Relative quantification (ΔΔCt) method was employed to analyze the data. Gene transcripts of islet-like aggregates were compared with human ductal adenocarcinoma cells (PANC-1). The qRT-PCR values are expressed as mean ± SEM.

**Table 1:** Primers sequences used in quantitative real-time PCR.

Name	Sequence	Annealing	products' Length
PDX-1-F	ATGGATGAAGTCTACCAAAGC	60	**159**
PDX-1-R	CGTGAGATGTACTTGTTGAATAG	PDX-1-R	
NGN3-F	AGAGAGCGTGACAGAGGC	60	**182**
NGN3-R	GCGTCATCCTTTCTACCG	60	
NKX2-2-F	AGTACTCCCTGCACGGTC	60	**103**
NKX2-2-R	GTCTCCTTGTCATTGTCCG	60	
GLUT2-F	TCACTGCTGTCTCTGTATTCC	60	**147**
GLUT2-R	TGCTCACATAACTCATCCAAG	60	
INS-F	GAACGAGGCTTCTTCTACAC	59	**143**
INS-R	ACAATGCCACGCTTCTG	59	
GCG-F	ACCAGAAGACAGCAGAAATG	59	**191**
GCG-R	GAATGTGCCCTGTGAATG	59	
PPY-F	GCCACACCAGAGCAGATG	61	**89**
PPY-R	TTGTGTCTTTTCCCATACCTAG	61	
Somatostatin-F	GGGAAGCAGGAACTGGC	63	**127**
Somatostatin-R	GCTCAAGCCTCATTTCATCC	63	
FOXa2-F	GGA GCG GTG AAG ATG GAA GG	68	**93**
FOXa2-R	CGG CGT TCA TGT TGC TCA C	57.1	
SOX17-F	CAA GAT GCT GGG CAA GTC	56.0	**90**
SOX17-R	TGG TCC TGC ATG TGC TG	55.0	

### Immunofluorescence analysis

Cells were seeded at a density of 1X10^5^ /well on an 8-well chamber slide and fixed in 4% paraformaldehyde (PFA) in PBS for 20 min at RT. Then the cells were permeabelized using 0.2% Triton X-100 for 5 min at room temperature and incubated overnight with primary antibody at 4°c. The primary antibodies were mouse anti-human insulin (1:100) (# ab7760, Abcam, Cambridge, MA, UK), mouse anti-human Glucagon (1:200) (#ab10988), mouse anti-human PDX1 (1:200) (#ab84987) and mouse anti-human Neurogenin3 (1:100) (#ab87108). After washing with PBST 3 times, the slides were incubated with fluorescence-labeled secondary antibody, including FITC-coupled goat anti-mouse IgG (1:50) (#AF8032, Razi Biotech, Iran) and Texas Red-labeled goat anti-mouse IgG (1:100) (ab5884) for 45 min at RT away from dark. Nuclear DNA was stained with 0.1μg/ml of blue-fluorescent 4', 6-Diamidino-2-phenylindole (DAPI) (Sigma) at 30°C for 5 min after washing with PBST 3 times. The slides were mounted with glycerol-PBS and visualized under a fluorescence microscope (Nikon.US). All experiments were repeated at least two times. In addition, pancreatic endocrine markers checked by western blot analysis.

### Western blotting

Cells were washed twice with ice-cold PBS and total protein was extracted from differentiated and undifferentiated hMSCs on day 21 after infection with radioimmunoprecipitation assay (RIPA) buffer containing 10mM Tris-HCl (PH = 8.0), 1% NP-40, 10% Glycerol, 0.1% SDS,1mM EDTA and 100mM Nacl with protease inhibitor cocktail (Roche Diagnostic, GmbH, Germany). 50μg of total protein was heated to 95°C for 5 min and separated by 15% sodium dodecyl sulfate-polyacrylamide (SDS-PAGE) gel and transferred to nitrocellulose membrane (Amersham Biosciences). The membrane was blocked for 2 hrs in 3% nonfat skim milk in Tris-buffered solution with 0.1% Tween 20 (TBST) and then incubated overnight at 4°C with rabbit anti-human Myotrophin primary antibody (#ab90918, Abcam, Cambridge, MA, UK), rabbit anti-human ONECUT2 primary antibody (#ab83206, Abcam, Cambridge, MA, UK) and rabbit anti-human-Granuphilin antibody (ab110519, Abcam, Cambridge, MA, UK). After washing with TBST, the membranes were incubated with polyclonal anti-rabbit horseradish proxidase-conjucated (HRP) secondary antibody (1:500) (#AP7181, Razi Biotech, Iran) for 1hr at room temperature and after 3 times of washing with TBST, bands were visualized using enhanced chemiluminescence (ECL) reagent (Ariyatous Biotech, Iran) according to the manufacturer’s instructions and were compared with β-actin. PANC-1cells were used as a positive control.

### Measurement of insulin and C-peptide secretion and content

hMSCs transfected with lentivirus carrying miR-375 gene and/or anti-miR-9, control cells with empty lentivirus and hMSCs without any treatment were plated at a density of 10^6^ cells per well in a 6-well plate and maintained in culture media for 21 days. After this time, cells were washed with PBS and incubated in freshly prepared Krebs-Ringer bicarbonate (KRB) buffer (120mM NaCl, 5mM KCl, 2.5mM CaCl2, 1.1 mM MgCl2, 25 mM NaHCO3 and 0.1% BSA) without glucose for 2 hrs. Then the cells were incubated for 2 hrs in KRB containing 0.5 mM l-isobutyl-3-methylxanthine (IBMX) (Sigma) and glucose in different concentrations. The suspension liquid was collected and stored at -70°C until being assayed. Insulin and C-peptide released into the medium were measured by an ultrasensitive enzyme-linked immunosorbent assay (ELISA) kit (#10-1132-01, #10-1141-01, Mecodia, Uppsala, Sweden) according to the manufacturer’s instructions. In addition to measurement of intracellular insulin and C-peptide, the cells were washed 3 times with PBS, extracted in 0.2ml acid alcohol (10% glacial acetic acid in absolute ethanol) at 4°C overnight, and then sonicated 3 times 15 sec each at 40–60 w, and centrifuged (13.000rpm, 15min, 4°C). Next, the supernatant was collected and frozen at -70°C until being assayed. Total protein concentration was determined by BCA protein assay system and 50μg of protein was used for detection of intracellular insulin and C-peptide in each well of ELISA kit. According to the kit, conversion factor for insulin was 1μg/ml = 23mu/l; 1mu/l = 6.0 pmol/l and for C-peptide 1μg/l corresponded to 331 pmol/l.

### Statistical analysis

The values are presented as the mean ± SEM. Results were analyzed by one-way ANOVA and Bonferroni’s post-hoc test was used for comparison of experimental groups with control by Graphpad Prism5 software. P-value less than 0.05 were considered statistically significant.

## Results and Discussion

### Isolation and characterization of undifferentiated hBM-MSCs

**Fig 1:**
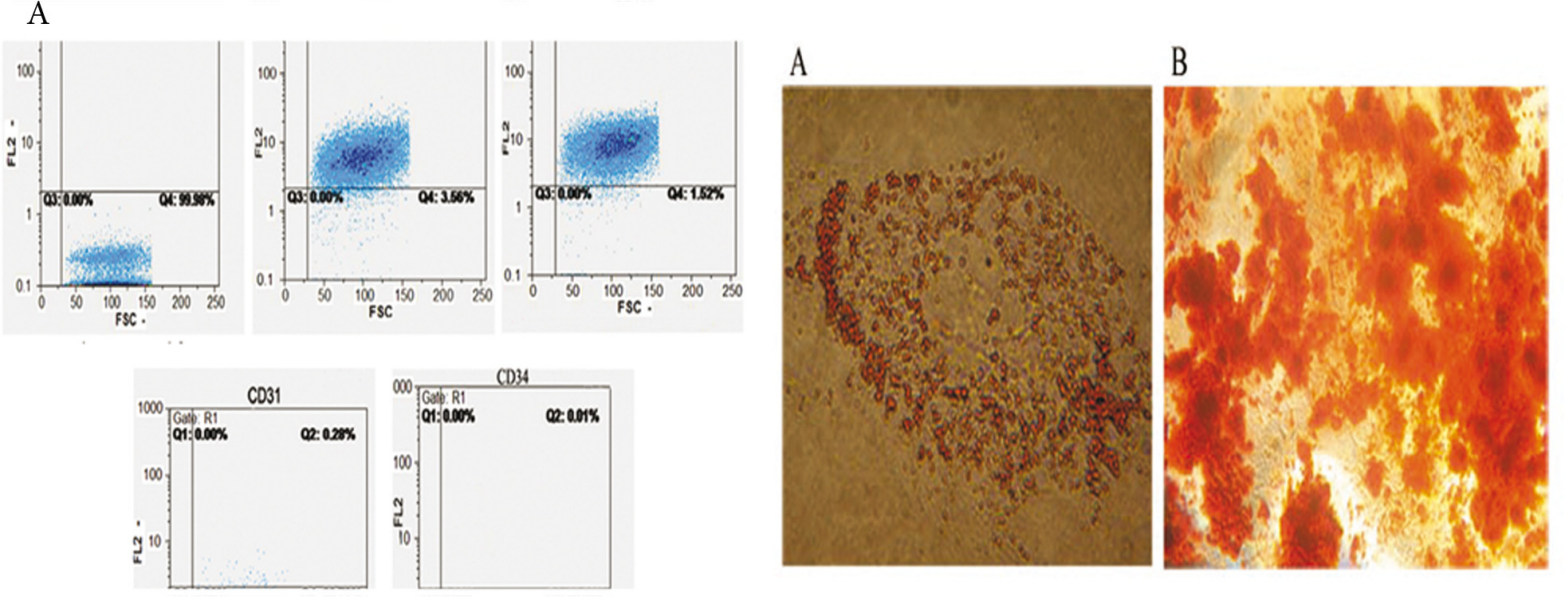
Characterization of undifferentiated hMSCs. (A) Flow cytometric analysis of hMSCs surface markers at passage 3, the cells was strongly positive for CD105 and CD90 while negative for CD34 and CD31. (B) The cells were exposed to lineage specific media and adipogenic and osteogenic differentiation was confirmed by (A) Oil-red (X100) and (B) Alizarin-red staining respectively (X100).

MSCs were isolated successfully from healthy human bone marrow sample and the purity rate of isolated cells was measured by flow cytometry. Flow cytometric analysis of surface phenotypes at passage 3 showed they were highly positive for CD105 (98.48%) and CD90 (96.44%) while negative for CD34 (0.01%) and CD31 (0.28%). MSCs were differentiated into mesodermal (adipocyte and osteocyte) lineage using adipogenic and osteogenic induction media. After 21 days of culture in osteogenic medium, osteogenic differentiation and mineral deposition in hMSCs were confirmed by Alizarin-red staining. Also, when the cells were induced with adipogenic medium for 14 days, adipocyte phenotype was observed by Oil-Red-O staining (Fig 1).

### Differentiation of lentiviral transduced hMSCs into IPCs

**Fig 2:**
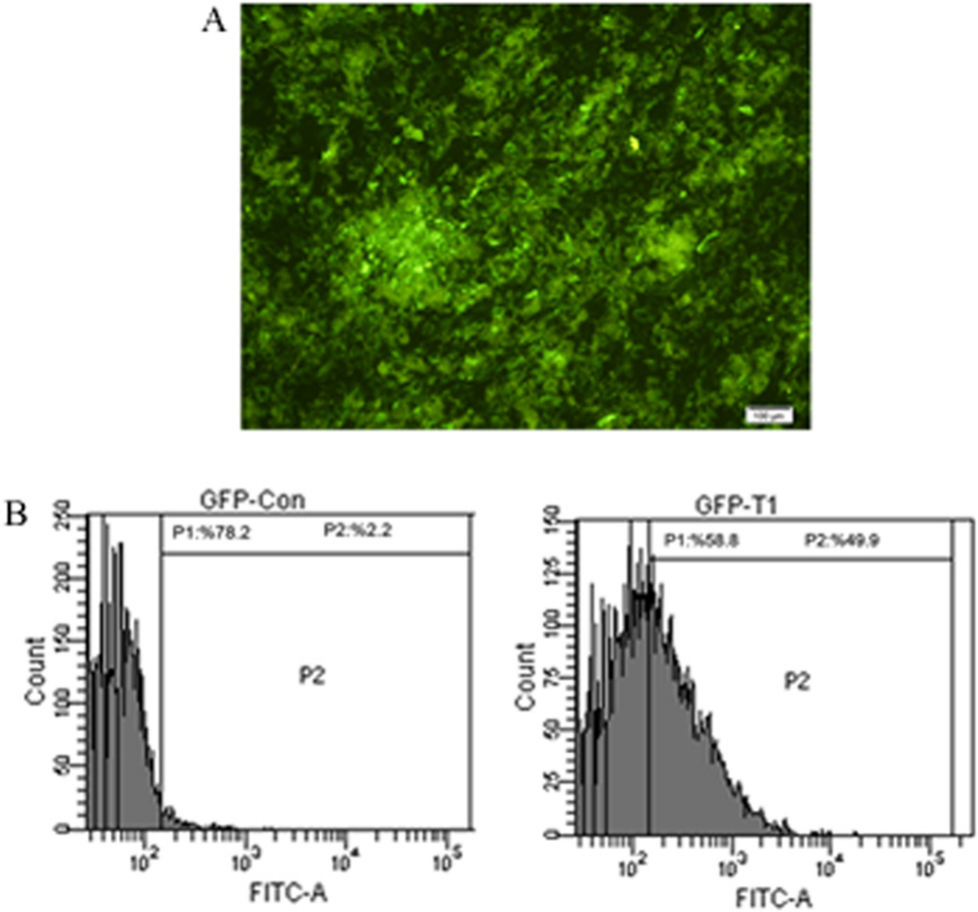
hMSCs purification and infection. (A) The results of miR-375 and anti-miR-9 transduction examined by fluorescent microscopy (X100). (B) Transduction efficiency of MSCs was about 50% in comparison to control MSCs as determined by flow cytometry. The experiment was repeated at least three times.

**Fig 3:**
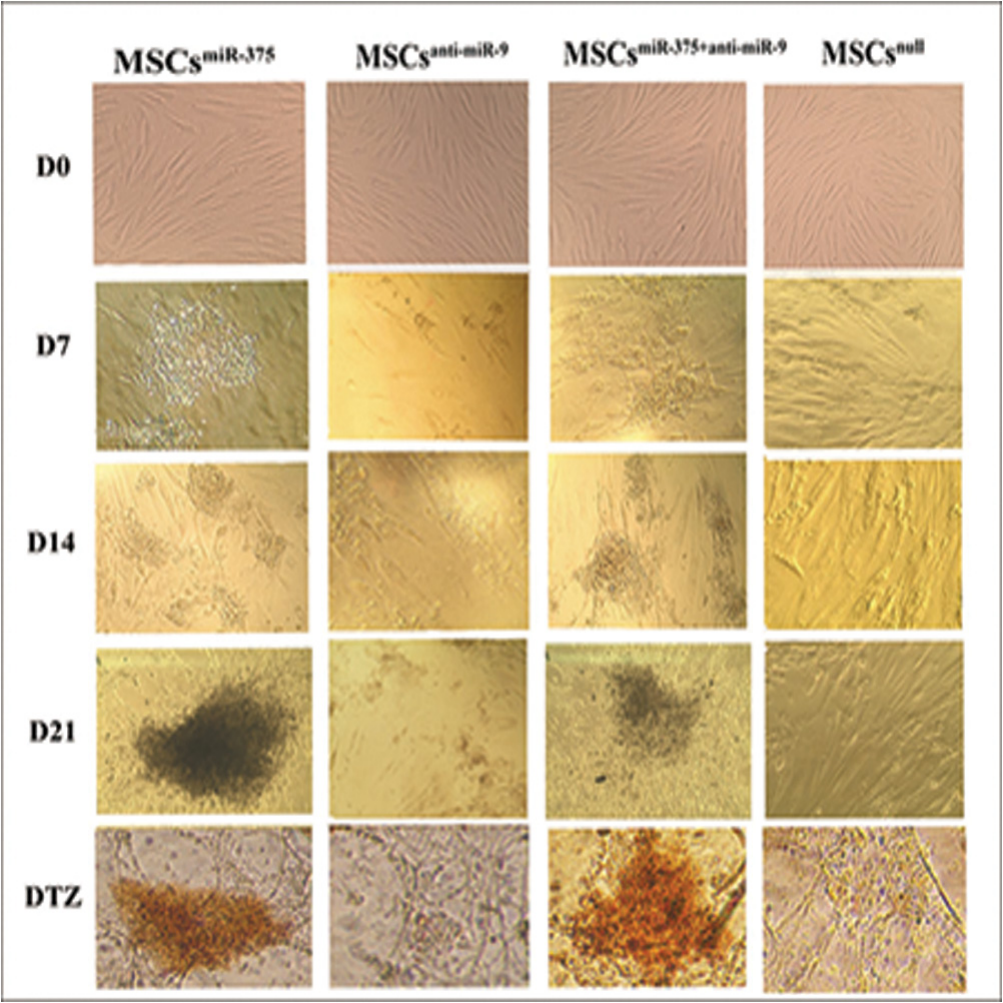
Morphological changes of hMSCs during differentiation and DTZ staining. Spindle shaped and fibroblast-like cells (D0) were induced to islet-like cluster formation by miR-375 and/or anti-miR-9 transduction in 21 days. After 7 days of induction, the cells became aggregate and some new islet-like clusters began to appear and after 14 days, the number of matured aggregates increased and at the end of day 21, some islet-like clusters were detached from plate and died. At this time, differentiated cells that were positive for DTZ staining appeared.

hMSCs at passage 3 were successfully transduced by lentiviral vectors. Transduction efficiency was checked each time by fluorescent microscopy and determined to be about 70% (Fig 2). Upon exposure to miR-375 and anti-miR-9 lentiviruses and serum free media, the adherent, spindle-like cells turned round and assembled together. Initial morphological changes of cells were observed 3 days after infections. During 2 weeks, the round cells became aggregate and some new islet-like clusters began to appear in MSCs^miR-375^ and MSCs^miR-375+anti-miR-9^ groups but they were not observed in MSCs^anti-miR-9^ (Fig 3). Therefore, the cells in MSCs^miR-375^ and MSCs^miR-375+anti-miR-9^ groups were selected for further studies. The culture of clusters was continued until maturation and further differentiation into islet-like structures. To assess the effect of over-expression of miR-375 and down-regulation of miR-9 on pancreatic islet-like differentiation, the researchers performed DTZ staining in the MSCs^miR-375^ and MSCs^miR-375+anti-miR-9^ groups. DTZ, specifically with binding to zinc ion in insulin molecules could identify clusters containing IPCs while undifferentiated cells were negative (Fig 3).

### Changes in transcript levels of miR-375 and miR-9 after infection

**Fig 4:**
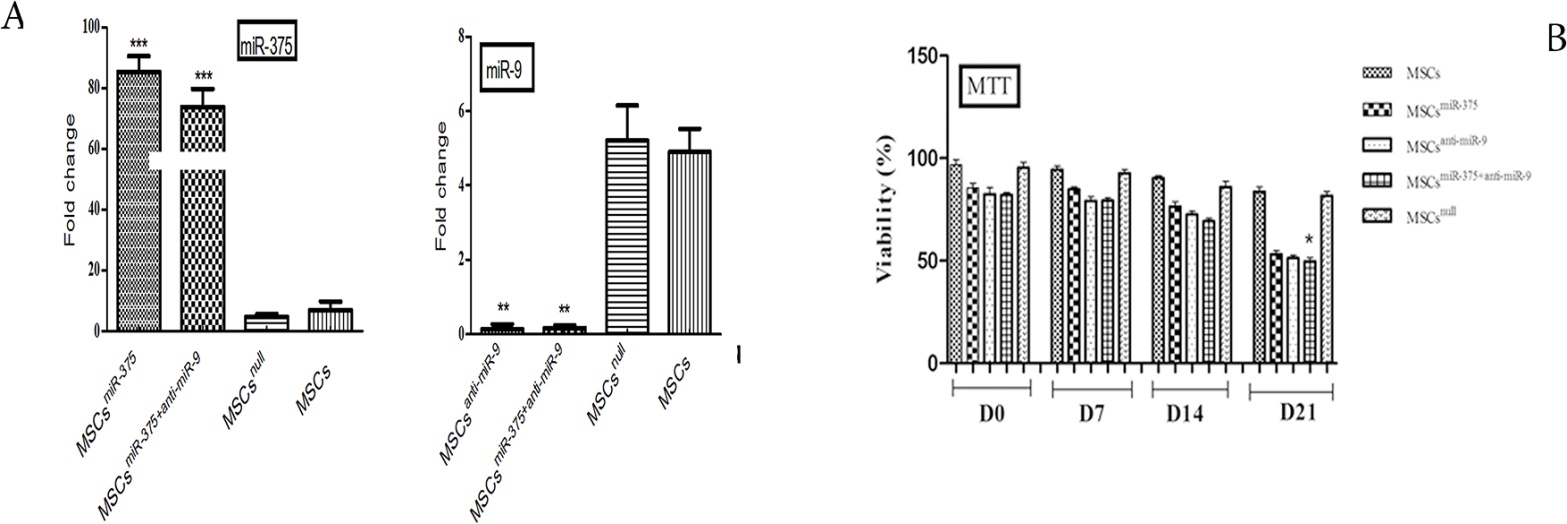
Changes in transcript levels of miR-375 and miR-9 after infection and viability assay. (A) Expression of miR-375 and miR-9 in test and control groups was measured by qRT-PCR on day 4 after transduction. All tests were performed in triplicate and data were presented as mean ± SEM. * *P* <0.05. (B) Proliferation ability of induced cells in all groups after 21 days, detected by MTT assay was slower than the MSCs control groups. * P <0.05

Expression levels of miR-375 and miR-9 on day 4 after transduction in tests and control groups by quantitative real-time PCR test indicated that the over- expression of miR-375 in MSCs^miR-375^ and MSCs^miR-375+anti-miR-9^ groups increased mature miR-375 expression by 85-fold and 87-fold as compared with MSCs^null^ and MSCs control groups respectively (p-value < 0.05 Fig 4A). Down-regulation of miR-9 in MSCs^anti-miR-9^ and MSCs^miR-375+anti-miR-9^ groups decreased mature miR-9 expression by 6-fold and 5- fold as compared with MSCs^null^ and MSCs control groups respectively (p-value < 0.05. It was found that miR-375 and miR-9 were strongly expressed in human BM-MSCs. As expected, when hMSCs cells were treated with empty lentiviruses, miR-375 and miR-9 expression levels showed no significant alteration as compared to MSCs control group. The proliferation ability of differentiated cells with lentivirus in MSCs^miR-375^ and MSCs^miR-375+anti-miR-9^ groups detected by MTT assay was slower than MSCs control groups (Fig 4).

### Expression of definitive endoderm markers in derived IPCs

**Fig 5:**
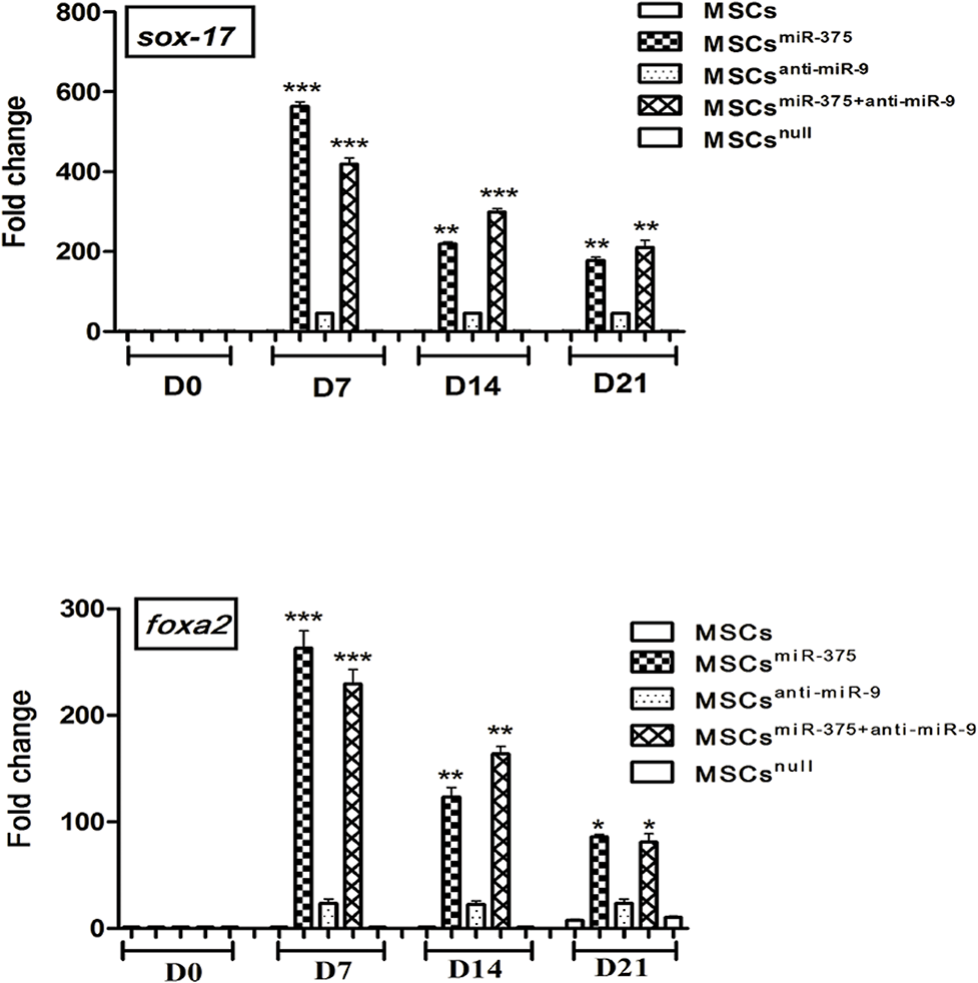
Expression of definitive endoderm markers. qRT-PCR analysis of definitive endoderm markers Sox-17 and HNF-3 beta/FoxA2 during differentiation on days 7, 14 and day 21 to islet-like clusters as compared with undifferentiated hMSCs (D0), the highest expression of both genes were detected on day 7. The expression levels were normalized to human GAPDH. The data are presented as mean±SEM. P <0.05.

The expressions of definitive endoderm markers like *SOX-17* and *HNF-3 beta/FoxA2* were detected by quantitative real-time PCR. The maximum levels of *SOX-17* in MSCs^miR-375^ and MSCs^miR-375+anti-miR-9^ groups were 563-and 419- fold and *HNF-3 beta/FoxA2* transcripts were 123- and 163- fold respectively and they were identified by day 7 in comparison to undifferentiated hMSCs (Fig 5).

### Expression of pancreatic endocrine genes in derived IPCs

**Fig 6:**
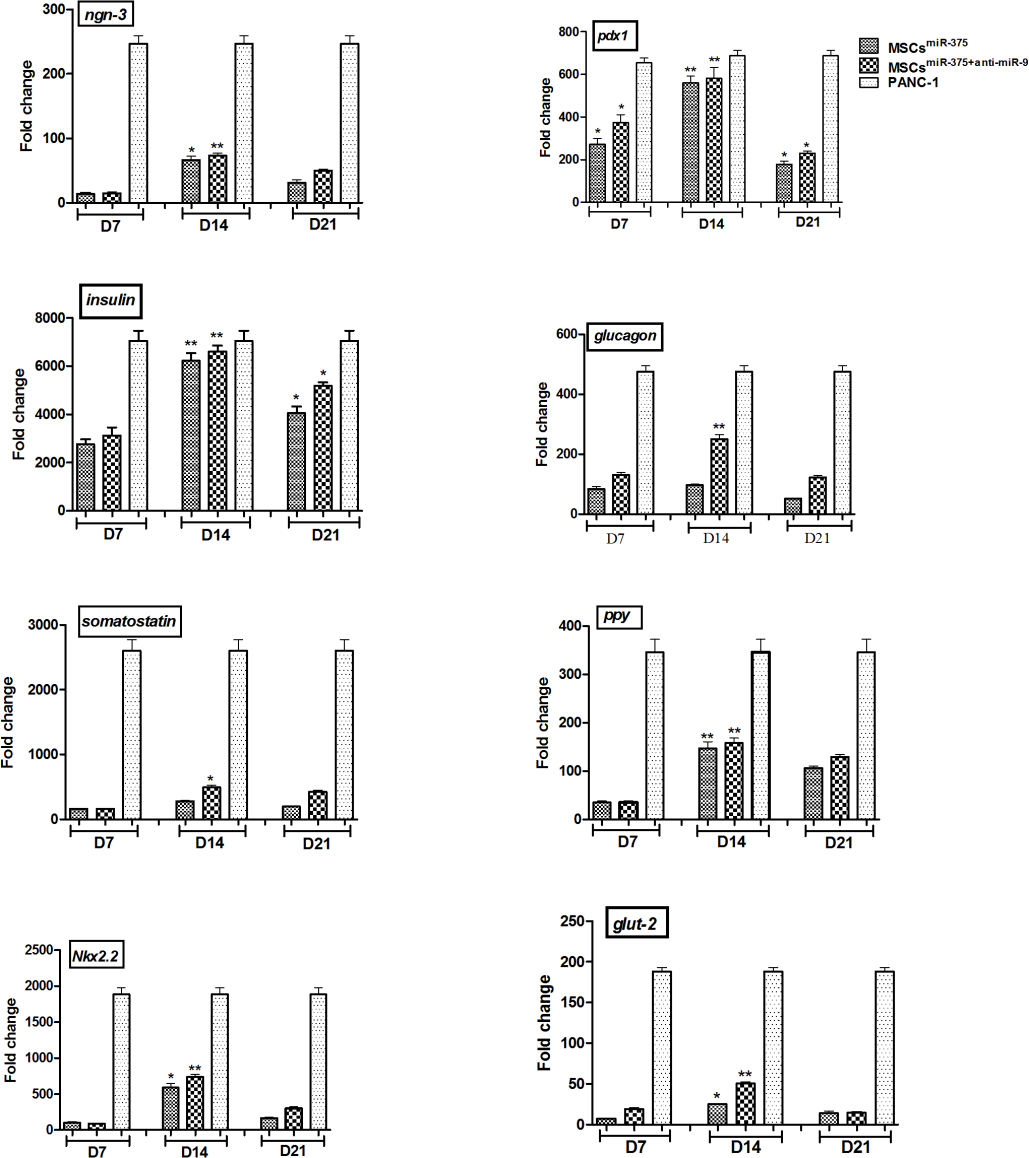
Expression of pancreatic endocrine genes in derived IPCs. The expression levels of pancreatic transcription factors such as *PDX1* and *Ngn3*, endocrine markers, *insulin*, *glucagon*, *somatostatin* and *PPY* and β cells specific genes like *Nkx2*.*2* and *GLUT2* were analyzed at each stage of differentiation into IPCs. Gene transcripts of IPCs were compared with undifferentiated hMSCs^null^ (negative control) and *PANC-1* cell line (positive control). Relative levels of gene expression were normalized to human *GAPDH*. Transcript value is shown in each graph as mean±SEM. * P <0.05.

As shown in Fig 6, mRNA expression in pancreatic development by key transcription factors such as *PDX1* and *Ngn3*, endocrine related marker genes including *insulin*, *glucagon*, *somatostatin and pancreatic polypeptide* and β cells specific genes like *Nkx2*.*2* as well as *GLUT2* were analyzed by quantitative real-time PCR on days 7, 14 and 21 post infections. The results revealed that all genes were expressed 7 days after infection and maximum levels of transcripts were detected on day 14 after induction and then gradually decreased in MSCs^miR-375^ and MSCs^miR-375+anti-miR-9^ groups. However, down-regulation of miR-9 in MSCs^anti-miR-9^ group had no obvious effect on expression of above mentioned genes. A significant up-regulation in the expression of pancreatic endocrine markers like *insulin*, *glucagon*, *somatostatin and pancreatic polypeptide* and major transcription factors involved in pancreas development such as *PDX1* and *Ngn3* and β cells specific genes like *Nkx2*.*2* as well as *GLUT2* were observed in mature D14 IPCs in MSCs^miR-375^ and MSCs^miR-375+anti-miR-9^ groups in comparison to undifferentiated hMSCs (p-value < 0.05). Taken together, these results further confirmed that miR-375 up-regulation contributes to islet-like aggregate differentiation of hMSCs and identifies this miRNA as one of the main factors that induces IPCs development.

### Effects of up-regulation of miR-375 and down-regulation of miR-9 on pancreatic markers

**Fig 7:**
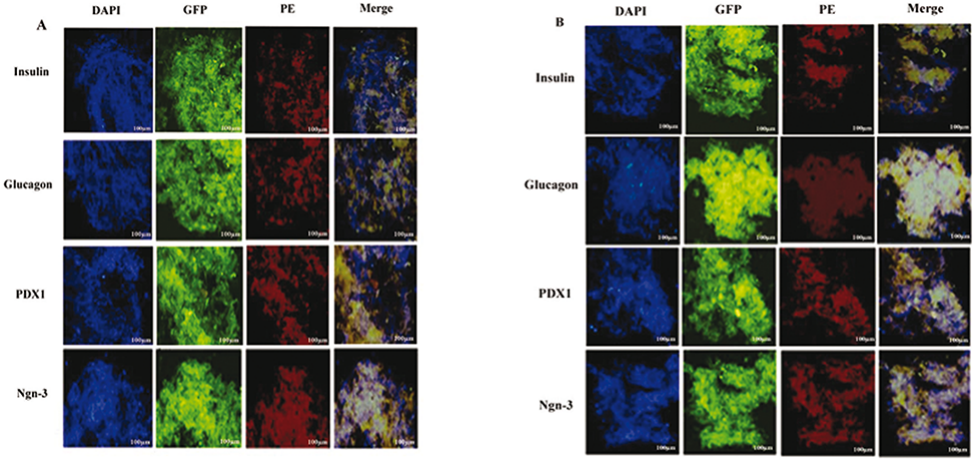
Immunocytochemical analysis of islet-like clusters. Immunofluorescence analysis detected nuclei localization of PDX1, Ngn3, and cytoplasmic localization of insulin, and glucagon in differentiated IPCs by (A) MSCs^miR-375^ and (B) MSCs^miR-375+anti-miR-9^ on day 21. Qunter-staining of nucleus (blue) was performed by DAPI. Images were obtained by a fluorescence microscope (X100).

**Fig 8:**
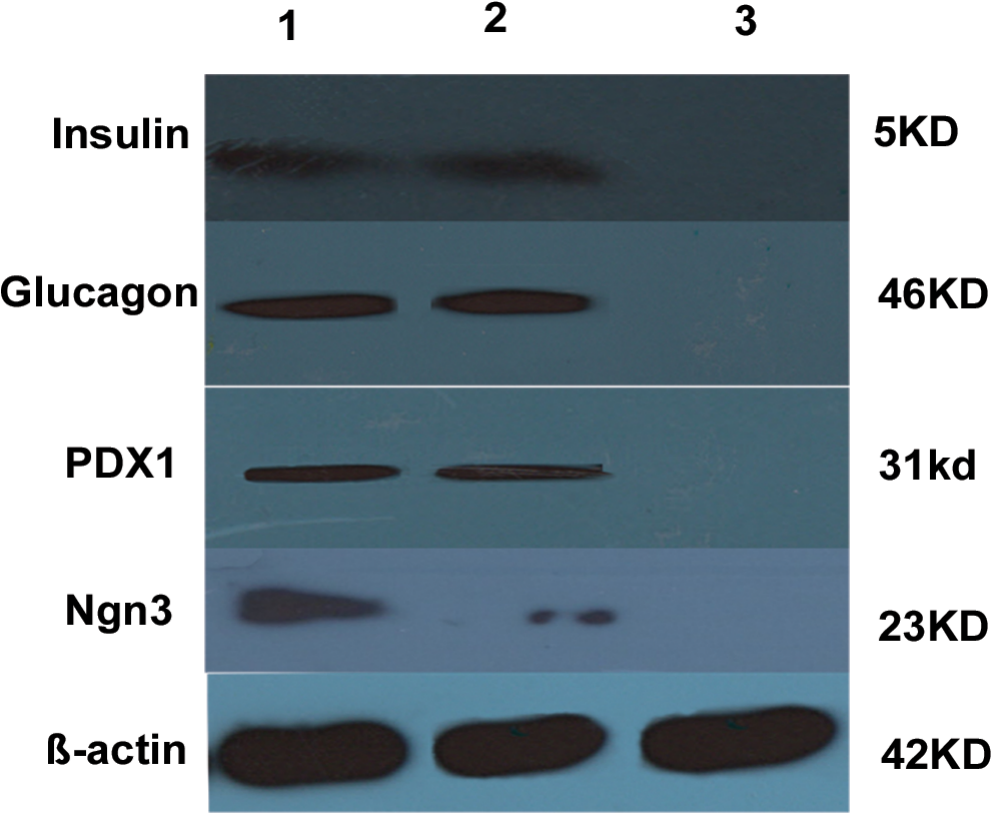
Detection of pancreatic endocrine proteins levels by western blot in IPCs. Western blot analysis detected expressions of insulin (5KD), glucagon (46KD), PDX1 (31KD) and Ngn3 (23KD) in differentiated IPCs (1) MSCs^miR-375^ and (2) MSCs^miR-375+anti-miR-9^ (3) MSCs^control^.

To determine whether up-regulation of miR-375 in MSCs^miR-375^ and down-regulation of miR-9 in MSCs^miR-375+anti-miR-9^ groups affected pancreatic islet cell differentiation, immunostaining of insulin, glucagon, PDX1 and Ngn-3 was performed. Immunofluorescence analysis detected nuclei localization of PDX1, Ngn3, and cytoplasmic localization of insulin and glucagon in differentiated IPCs on day 21 in both groups. Qunter-staining of nucleus (blue) was performed by DAPI (Fig 7). In addition western blot analysis also confirmed pancreatic endocrine expressions after induction in MSCs^miR-375^ and MSCs^miR-375+anti-miR-9^ groups (Fig 8). Taken together, the expression of these important pancreatic transcription factors and hormones strongly indicated that both recombinant lentiviruses are able to promote IPCs differentiation of the MSCs without addition of any stimulatory growth factors and cytokines.

### Western blot analysis and the role of miR-375 in Myotrophin protein expression

**Fig 9:**
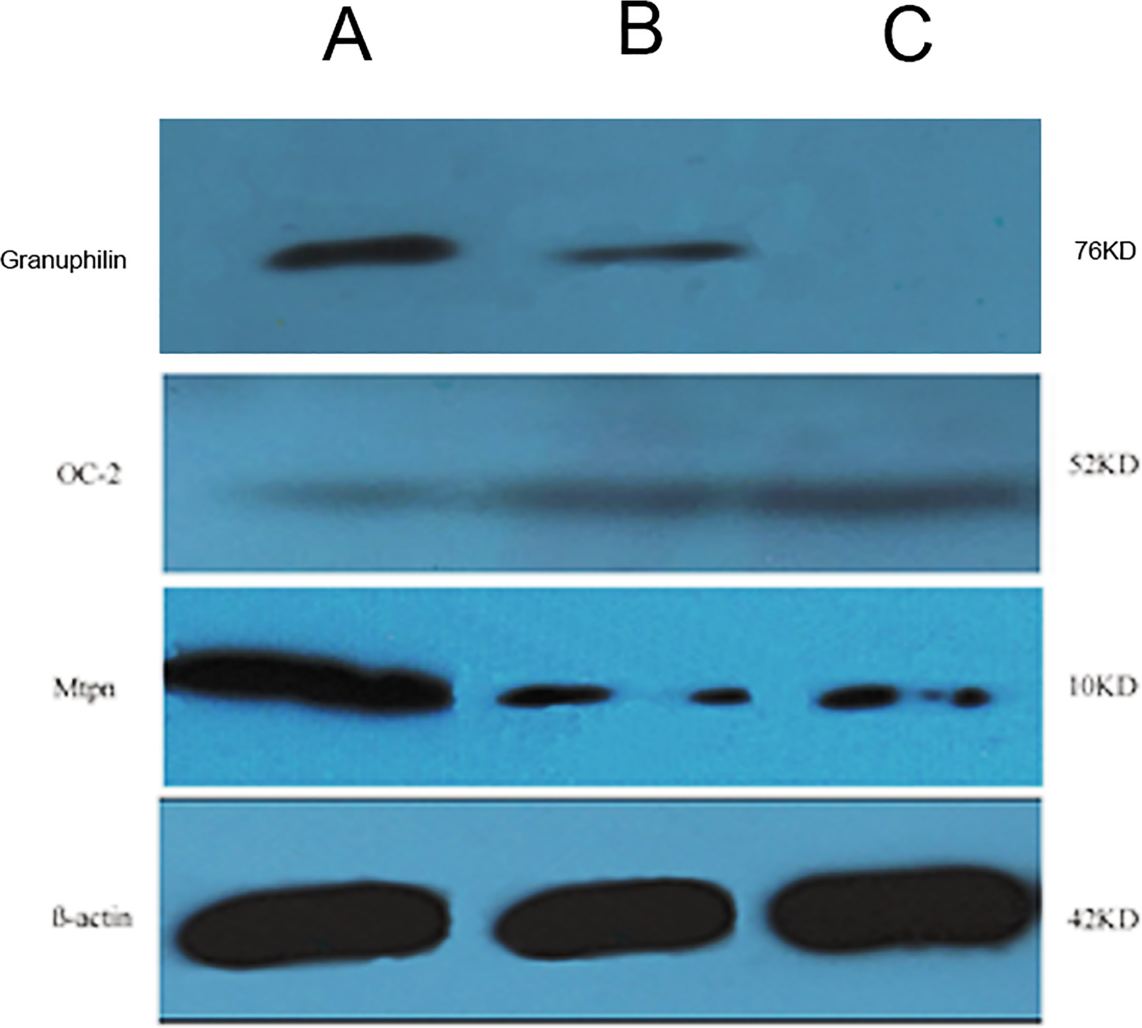
Detection of Mtpn and OC-2 proteins levels by western blot in IPCs. Granuphilin: (A) MSCs^miR-375+anti-miR-9^ (B) MSCs^miR-375^ (C) Control hMSCs, OC-2: (A) Control hMSCs (B) MSCs^anti-miR-9^ (C) MSCs^miR-375+anti-miR-9^. Mtpn: (A) Control hMSCs (B) MSCs^miR-375^ (C) MSCs^miR-375+anti-miR-9^. Protein level of Mtpn was markedly reduced after miR-375 up-regulation. However, a protein level of OC-2 was increased following anti-miR-9 infections after 21 days.

The protein expressions of target genes, Myotrophin and Onecut-2, were also detected after lentiviral mediated differentiation into IPCs in MSCs^miR-375^ and MSCs^miR-375+anti-miR-9^ groups. Western blot results indicated that protein level of Myotrophin was markedly reduced in MSCs infected with miR-375 lentivirus. However, Onecut-2 protein expression was higher in MSCs infected with anti-miR-9 lentivirus (Fig 9).

### Effect of miR-375 over-expression and miR-9 down-regulation on differentiated IPCs functions

**Fig 10:**
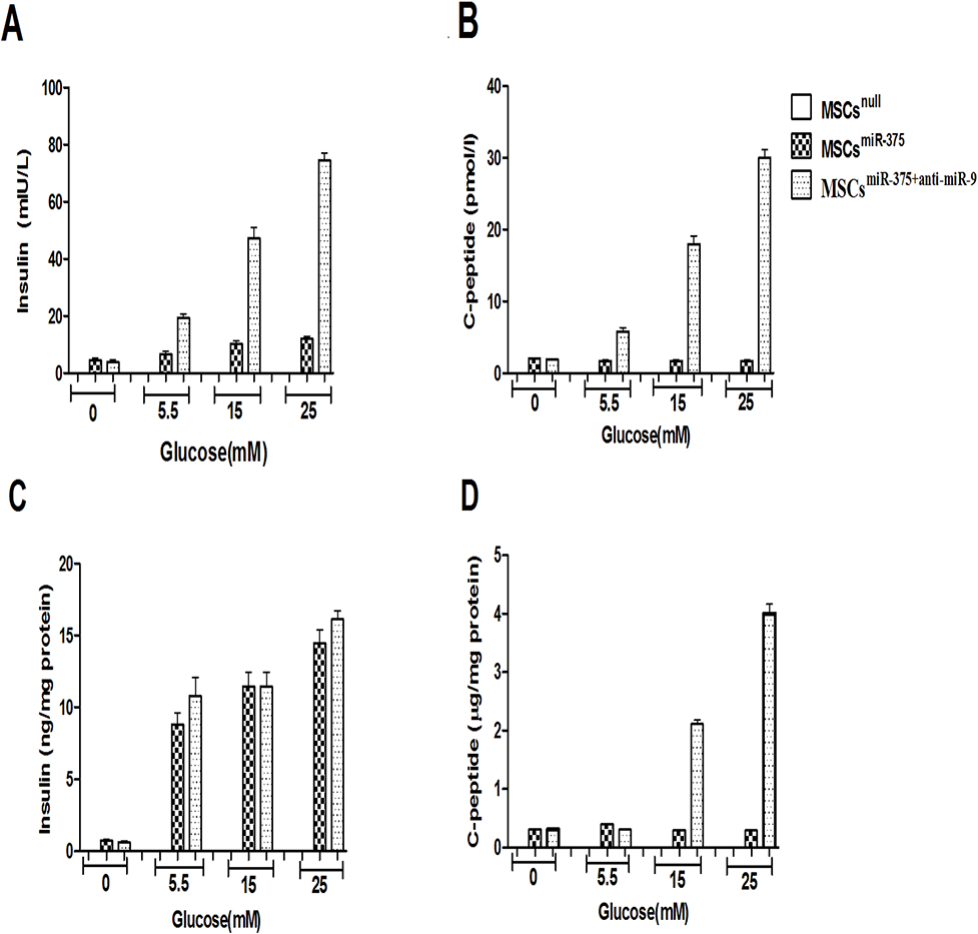
In vitro glucose assay in derived IPCs. (A) Insulin secreted in cultured media in response to various concentrations of glucose from 5.5 to 25 mM; (B) C-peptide released in culture media; (C) Intracellular insulin content in each concentration of glucose that normalized with total cellular protein; (D) Intracellular C-peptide content in different concentrations of glucose that normalized with total cellular protein. Significant amounts of insulin and C-peptide were obtained in MSCs^miR-375+anti-miR-9^ group. The data are presented as mean ± SEM. P <0.05.

The researchers employed glucose challenge test for future examination of the functionality of induced cells to secret insulin and C-peptide in response to various concentrations of glucose. A number of different stimulators were recognized that have regulatory functions in insulin secretion so the researchers used l-isobutyl-3-methylxanthine (IBMX) to increase intracellular cyclic-AMP (cAMP) levels and inhibit cAMP phosphodiesterase to increase insulin and C-peptide secretion [24]. Differentiated MSCs with miR-375 lentivirus (MSCs^miR-375^ group) didn’t have any response to various concentrations of glucose and no insulin and C-peptide were detected in this group (p-value > 0.05). However, in derived IPCs mediated by miR-375 over-expression and then miR-9 down-regulation (MSCs^miR-375+anti-miR-9^ group), insulin and C-peptide secretion and content were enhanced by different concentrations of glucose from 5.5 to 25mM. The cells in 0, 5.5, 15 and 25 mM glucose, secreted 4 ± 0.6 mu/l, 19.46 ± 3.1 mu/l, 47.3 ± 11.3 mu/l and 74.3 ± 14.29 mu/l insulin (n = 12) and the amounts of C-peptide were 1.86 ± 0.45 pmol/l, 5.7 ± 0.98 pmol/l, 18± 11.34 pmol/l and 30± 14.38 pmol/l (n = 12) respectively. Moreover, the insulin and C-peptide content in differentiated cells were measured. The values for insulin in 0, 5.5, 15 and 25 mM glucose concentrations in MSCs^miR-375^ group were 0.77 ± 0.9 ng/mg, 8.8 ± 1.32 ng/mg, 11.48 ± 4.6 ng/mg, 14.46 ± 5.36 ng/mg and for C-peptide were 0.3± 0.16 μg/mg, 0.4± 0.2 μg/mg, 0.29± 0.26 μg/mg,0.3± 0.18 μg/mg and values in 0, 5.5, 15 and 25 mM glucose for MSCs^miR-375+anti-miR-9^ group were 0.64± 0.5 ng/mg, 10.8 ± 3.9 ng/mg, 11.46 ± 6.9 ng/mg, 16.13± 9.9 ng/mg for insulin and 0.32 ± 0.19 μg/mg, 0.3 ± 0.16 μg/mg, 2.1 ± 0.24 μg/mg and 3.97± 1.4 μg/mg respectively. Thus, the obtained results indicated that maturated IPCs in MSCs^miR-375+anti-miR-9^ group unlike MSCs^miR-375^ could produce significant amount of insulin as compared to undifferentiated hMSCs (Fig 10).

Bone marrow mesenchymal stem cells (BM-MSC) reside in bone marrow and are multipotent and can differentiate into lineages of mesenchymal tissues, such as bone [25], cartilage [26], tendon [27], muscle [28], adipocytes [29], chondrocytes [30], osteocytes [29]. MSCs could differentiate into endodermal and epidermal cells, such as vascular endothelial cells, neurocytes, and hepatocytes [31]. Mesenchymal stem cells derived from bone marrow are now in clinical trials after being examined in terms of safety and efficacy in animal models, so the cells are MSC a promising source in cellular and gene therapies. Recent studies have shown that miRNAs are involved in various physiological events, such as developmental timing [32, 33], apoptosis [34], cell proliferation [34], muscle growth [35] and organ function [6, 36, 37]. Interestingly, miRNAs have been detected in normal and pathological conditions including, cancer [38, 39], neurological disorders [40, 41], inflammation [42] and diabetes [43, 44]. They are involved in self-renewal and differentiation of embryonic stem cells, but their exact mechanism in MSCs has been poorly understood. In recent studies, the over-expression of miR-375 in murine pancreatic β-cell line (MIN6) resulted in a decrease in glucose dependent insulin secretion. On the other hand, functional knockdown of miR-375 in mice by using 2'-o-methyl antisense oligonucleotides had a converse effect and increased the insulin secretion in response to glucose [45]. The role of miR-375 in pancreas development and islet integrity was revealed from knockdown experiments during zebrafish embryonic development [14]. miR-375 was involved in insulin secretion by Mtpn targeting. Mtpn is a transcriptional activator of nuclear factor- kappaB (NF-kappaB) and the activation of this factor is associated with glucose dependent insulin secretion [15]. On the other hand, miR-9 is a negative regulator in glucose stimulated insulin secretion [16]. miR-9 regulates insulin secretion by targeting OC-2 mRNA and down-regulates its expression in insulin producing cells [18]. In this study, the researchers provided in vitro evidence for the differentiation of hBM-MSCs into IPCs by the influence of miRNAs. The researchers tested whether miR-375 and/or anti-miR-9 lentiviruses infected cells were able to produce, store and secret insulin. Despite insulin gene and protein expression, no response to glucose in derived IPCs by miR-375 lentivirus was detected. By contrast, insulin content and secretion were enhanced by increasing glucose concentrations from 5.5 to 25mM in MSCs^miR-375+anti-miR-9^. The results show, for the first time, that whereas derived IPCs by over-expression of miR-375 alone in MSCs were capable to express insulin and other endocrine specific transcription factors, the infected cells lacked the machinery to respond to glucose. Insulin hormone was accumulated in differentiated cells without the ability to secrete. According to the findings, over-expression of miR-375 in MSCs impaired glucose dependent insulin secretion by down-regulating Mtpn, a protein that is involved in modulation of insulin vesicle exocytosis from β-cells and which results in a defect in insulin exocytosis. The researchers showed that lentiviral mediated down-regulating of miR-9 in MSCs didn’t have any effect on differentiation and insulin secretion. Moreover, differentiated MSCs by miR-375 and anti-miR-9 lentiviruses simultaneously secreted insulin and c-peptide in a glucose-induced manner. Specifically, it was shown that inhibition of miR-9, which is expressed in differentiated IPCs, led to glucose-induced insulin secretion by up-regulation of the transcription factor OC-2 and therefore results in a decrease in granuphilin/Slp4 protein that has important role in negative control of insulin exocytosis. It was reported for the first time that miR-375 has essential role in MSCs differentiation into IPCs, while anti-miR-9 lentiviruses separately didn’t have any effect on MSCs differentiation into IPCs and subsequently insulin secretion. Simultaneous over-expression of miR-375 and down- regulation of miR-9 had synergistic effect on MSCs differentiation and insulin secretion in a glucose-regulated manner. This is the first time that transdifferentiation was induced by manipulating only the levels of miRNA without the use of transcription factors or cocktail media. Moreover, although the roles of miR-375 and miR-9 are well known in pancreatic development and insulin secretion, the use of these miRNAs in transdifferentiation was never demonstrated. However, future studies are required to develop in vivo delivery technique for these miRNAs. These findings highlight miRNAs functions in stem cells differentiation and suggest that they could be used as therapeutic tools for gene-based therapy in diabetes mellitus.

## Conclusion

In conclusion, we have shown that hMSCs can be induced by miR-375 and/or anti-miR-9 to differentiate into mature islet like clusters. miR-375 up regulation alone may not responsible for the cell responsive to glucose challenge. On the other hand suppression of miR-9, that has inhibitory role in insulin secretion, could an effective way to give glucose response in vitro. These findings highlight the importance of miRNAs in stem cell development and differentiation in vitro. Although additional examinations including in vivo studies needed to validate our finding for cell replacement therapy in insulin dependent diabetes.
